# Developing Placebos for Clinical Research in Traditional Chinese Medicine: Assessing Organoleptic Properties of Three Dosage Forms (Oral Liquid, Capsule and Granule)

**DOI:** 10.3389/fphar.2021.673729

**Published:** 2021-06-17

**Authors:** Mengli Xiao, Jiake Ying, Yang Zhao, Qingna Li, Yingpan Zhao, Rui Gao, Fang Lu

**Affiliations:** ^1^ NMPA Key Laboratory for Clinical Research and Evaluation of Traditional Chinese Medicine, Xiyuan Hospital of China Academy of Chinese Medical Sciences, Beijing, China; ^2^ National Clinical Research Center for Chinese Medicine Cardiology, Xiyuan Hospital of China Academy of Chinese Medical Sciences, Beijing, China; ^3^ Department of Gastroenterology, Xiyuan Hospital of China Academy of Chinese Medical Sciences, Beijing, China

**Keywords:** traditional Chinese medicine, placebo, simulation effect, evaluation, organoleptic properties

## Abstract

**Background:** The successful application of randomized, double-blind placebo-controlled studies requires maximum blinding. Organoleptic properties of the placebo should be similar to the drug, making it difficult to distinguish between the two. The uniqueness of traditional Chinese medicine (TCM) preparations makes it challenging to prepare placebo. Evaluation of the TCM placebo simulation effect can determine whether the preparation of placebo can be genuinely blind in clinical trials. There is still a lack of well-established methods to evaluate TCM placebos. Hence, this study aimed to explore the evaluation methodology of TCM placebo simulation.

**Methods:** An independent evaluation method and three comparative evaluation methods were proposed, and three dosage forms (oral liquid, capsule, and granule) were tested. The independent evaluation, in which each person was given an experimental drug or a placebo, gave an overall assessment of organoleptic properties in a blind state. We comparatively evaluated the similarity in organoleptic properties between the experimental drug and placebo. According to different distribution methods, we divided comparative evaluation methods into three. In method 1, the evaluator was given the experimental drug and placebo and was told that there must be a placebo among them. In method 2, each evaluator was randomly assigned to the combination group or two investigational drugs group. In method 3, the evaluator was assigned to a set of three coded samples, numbered by random three-digit numbers, each different, two of which were identical, and the two samples were equally frequent.

**Results:** In the independent evaluation, there was no difference between TCM placebo and experimental drugs in a blind state at the level of *p* = 0.05. Even though the comparative evaluation methods enabled identification of potential differences between the two samples, methods 2 and 3 were better than method 1 in eliminating psychological factors. Also, in method 3, the completely random method combined with the blind method eliminated the subjectivity and objectivity bias and improved the experiment’s credibility compared with the previous two methods.

**Conclusion:** Regardless of the methods that could evaluate the placebo’s simulated effect in actual clinical trials, we suggest that independent evaluation and comparative evaluation (method 3) should be combined to reflect better whether the placebo is truly blind.

## Introduction

Masking of participants and researchers has long been used in randomized clinical trials (RCTs) to eliminate the potential impacts of nondrug effects, including the natural course of the disease; the evaluator and researchers; and subjective factors in treatment, diagnosis, and clinical assessment (Jamshidian et al., 2014; Dube et al., 2007). In the past 40 years, at least 17,000 RCTs have been conducted in China to assess the efficacy and safety of traditional Chinese medicine (TCM), including Chinese herbal medicine, acupuncture, massage, moxibustion, Qigong, and other therapies, most of which are related to Chinese herbal medicine (Wang et al., 2007). Regardless of the increasing number of TCM studies, their reliability has been challenged because of the lack of rigorous evidence (Wang et al., 2007; Teschke et al., 2015; Liu et al., 2015). Inadequate randomization, insufficient sample sizes, and the lack of proper blinding make the research results vulnerable to the selection, reporting, and assessor bias.

A more recent review of TCM uncovered that many studies often lacked true blindness (Teschke et al., 2015). To meet the requirements for blinding, it is necessary to prepare a convincing TCM placebo, and these must be similar to the investigational drug in terms of visual attributes, dosage form features, and smell/taste attributes. Compared with western medicine placebo drugs, the unique odor, taste, and color of TCM preparations make it challenging to prepare placebo. At present, there are two forms of placebo preparation: no pharmacodynamic components and low-dose pharmacodynamic components. The placebo prepared by the simple excipient method placebo does not contain pharmacodynamic components. Generally, it uses flavorants (edible additives), colorants (edible pigments), and volatiles (agents to simulate a placebo) (Lu et al., 2018). The other is to use the drug with excipients in a low proportion. It is feasible to dilute the drug substance by 10 or 20 times, use it as a placebo, or use materials with certain pharmacological activities unrelated to the investigational drug’s effect to prepare the placebo (Nakaya et al., 2003). To explore whether the organoleptic properties of the placebo are exactly the same as those of experimental drugs, it is imperative to evaluate the TCM placebo’s simulation effect. Nevertheless, there is a lack of recognized evaluation methods and standards for TCM placebos (Wang et al., 2014).

At present, there are mainly two clinical evaluation methods: artificial and objective evaluation. For artificial evaluation, some researchers have proposed a placebo quality checklist (Brinkhaus et al., 2008), but this has never been appropriately validated. Some Chinese researchers have proposed to allow healthcare professionals, pharmaceutical companies, and patients to score placebos, and the results should be used to determine whether the placebo simulation is successful (Tang et al., 2009; Wang et al., 2011; Jin et al., 2014a; Song et al., 2014; Yang, 2014; Sun et al., 2019; Yan and Zhang, 2020). For objective evaluation, some researchers have begun to use intelligent sensory analysis to evaluate placebo quality, including visual sensors, electronic tongue sensors, and other technologies. These are used to assess placebo consistency with the investigational drug in organoleptic properties, and they can transform subjective evaluation reports into objective data to standardize evaluations (Luo, 2012; Jin et al., 2014b; Liu et al., 2014; Fan, 2018).

Manual placebo evaluation can maximize the simulation of the patient judgment of drugs in the real clinical environment. Here, we proposed four methods for evaluating the simulated effect of placebo in TCM, including an independent evaluation method and three comparative evaluation methods. In the independent evaluation, only one of the two drugs, including experimental drug and placebo, was given to the evaluator. In contrast, in the comparative evaluation, the evaluator was given multiple boxes of drugs, including various possibilities. To fully explore whether the four evaluation methods identified and evaluated the simulation effect of TCM preparations stably, we took three commonly used TCM preparations (oral liquid, granule, and capsule) as an example to conduct the simulation effect evaluation test. We provided our insights and considerations for evaluating the manufacture and simulated effects of TCM placebos.

## Materials and Methods

### Evaluation of Different Dosage Forms of Traditional Chinese Medicine Placebo

This study was intended to evaluate three different dosage forms of TCM placebo used in clinical studies and the following were in each: 1) Fufang Ejiao Syrup (FFEJJ) oral solution (batch number: Z37021371); this product is a brown to dark brown liquid, sweet in taste, and comes in 20 ml vials. The ingredients and contents in placebo FFEJJ oral solution (20 ml) are listed in Table 1. Shandong Dong-e E-Jiao Co., Ltd., produced both the investigational drug and placebo. 2) Zhizhu Kuanzhong (ZZKZ) capsules (batch number: Z20020003); this product is light grayish brown, slightly bitter, salty and comes in 0.43 g/grains. The ingredients and contents in placebo ZZKZ capsules (0.43 g) are listed in Table 2. The investigational drug and placebo were produced by Langzhi Group Shuangren Pharmaceutical Co., Ltd., 3) Billing Weitong (BLWT) granules (batch number: Z19990069); this product is brown to tan granules with a bitter taste and comes in 5 g/bags. Ingredients and content contained in each placebo sachet (5 g) of BLWT granules are listed in Table 3. Both the investigational drug and placebo were prepared by Yangtze River Pharmaceutical Group Jiangsu Pharmaceutical Co., Ltd.

**TABLE 1:** Ingredients and their contents of FFEJ placebo in each placebo bottle (20 ml).

Ingredients	Content	Role(s)
Gelatina nigra syrup	0.9 ml	Equivalent to 5% of the original formula
Caramel pigment	0.8 g	Pharmaceutical excipient/food additive
Stevioside	0.025 g	Pharmaceutic adjuvant

**TABLE 2:** Ingredients and their contents of ZZKZ placebo in each placebo grain (0.43 g).

Ingredients	Proportion	Role(s)
Microcrystalline cellulose	1	Filler (no pharmacological activity)
Starch	2	Filler (no pharmacological activity)
Magnesium stearate	0.5%	Lubricant (no pharmacological activity)
Food-grade pigment solution	--	Food additive

**TABLE 3:** Ingredients and their contents of BLWT placebo in each placebo pack (5 g).

Ingredients	Content (g)	Proportion (%)	Role(s)
Dextrin	3.19	63.8	Pharmaceutic adjuvant
Sucrose	1.0	20.0	Pharmaceutic adjuvant
BLWT fine powder	0.375	7.5	Equivalent to 7.5% of the original formula
Povidone K30	0.25	5.0	Pharmaceutic adjuvant
Caramel	0.125	2.5	Pharmaceutical excipient/food additive
Low-substituted hydroxypropylcellulose	0.06	1.2	Pharmaceutic adjuvant

### Evaluation Methods of Placebo Simulation Effect of Traditional Chinese Medicine

The placebo should be completely consistent with the tested TCM in terms of appearance, color, odor, taste, packaging, usage, and dosage, so it was necessary to evaluate whether the placebo successfully mimicked the investigational drug by independent assessment and comparative effectiveness evaluation. Independent evaluation was required to determine if the actual medication was simulated. The evaluator determind whether a sample in the investigational drug or placebo was an investigational drug under a blinded state; for comparative effect evaluation, they evaluated the similarity of the investigational drug and placebo at different levels such as appearance texture, color, odor, and taste.

#### Independent Evaluation

Three dosage forms of FFEJJ oral solution, ZZKZ capsules, BLWT granules, and their placebos were evaluated. The investigational drug or placebo was randomly distributed to 20 evaluators (*n* = 10 each). The evaluators were randomly selected from the target evaluator for which the drug was acting. The evaluator made an overall assessment of the possibility that the sample was the investigational drug under a blinded state. There were two evaluation options: probably an investigational drug or probably a placebo. After unblinding, judgment accuracy was compared between the placebo and the investigational drug groups to determine whether there was a difference between them.

For the qualification criteria of the placebo simulation effect, the methods listed in Table 4 were adopted. Twenty evaluators (10 each for placebo and investigational drug) were used to calculate the difference in the proportion of the investigational drug and placebo judged as the investigational drug in the blinded state (*p *< 0.05). Assuming that the numbers of the evaluators who judged investigational drug as the investigational drug were 5, 6, 7, 8, 9, and 10, the cut-off value of the number of the evaluators who judged the placebo as the investigational drug varied from 0 to 5, indicating a significant difference at the 0.05 level. That is, when the number of evaluators was 20, and 5 evaluators judged the investigational drug as the investigational drug, the number of the evaluators who judged placebo as the investigational drug was at least 1, indicating there was no difference in the proportion of the evaluators who judged the placebo and investigational drug as the investigational drug in the blinded state at the 0.05 level. When all evaluators judged the investigational drug as the investigational drug, the number of the evaluators who judged the placebo as the investigational drug was at least 6, which means there was no difference in the proportion of evaluators who judged the placebo and the investigational drug as the investigational drug in the blinded state at the 0.05 level.

**TABLE 4:** Cut-off values of the difference in the number of evaluators who judged the investigational drug and placebo as the investigational drug at the 0.05 level.

	No. of evaluators = 20 (50/50 received investigational drug and placebo)		No. of evaluators = 40 (50/50 received investigational drug and placebo)	
	Investigational drug	Placebo	Investigational drug	Placebo
Investigational drug	10	0	20	0
Placebo	5	5	15	5
Investigational drug	9	1	18	2
Placebo	3	7	11	9
Investigational drug	8	2	16	4
Placebo	2	8	9	11
Investigational drug	7	3	14	6
Placebo	1	9	6	14
Investigational drug	6	4	12	8
Placebo	0	10	4	16
Investigational drug	5	5	10	10
Placebo	0	10	3	17

Comparably, if the number of evaluators was increased to 20 each for placebo and the investigational drug, with an assumption that the numbers of patients judged the investigational drug as the investigational drug were 10, 12, 14, 16, 18, and 20, and there was a difference in the proportion of the evaluators who judged both the investigational drug and placebo as the investigational drug in the blinded state (*p*< 0.05), the cut-off value of the number of evaluators who judged the placebo as the investigational drug varied from 3 to 15, suggesting a significant difference at the level of 0.05. That is, when 10 evaluators judged the investigational drug as the investigational drug, the number of evaluators who identified the placebo as the investigational drug was at least 4, which suggested there was no significant difference at the 0.05 level. When all evaluators judged the investigational drug as the investigational drug and the number of evaluatiors who thought the placebo was the investigational drug was at least 16, it was considered that there was no difference in the proportion of the evaluators who judged the investigational drug in the blinded state at the 0.05 level.

#### Comparative Evaluation

##### Method 1

All 20 evaluators were distributed an investigational drug and a placebo and the evaluators were told that one of them was a placebo, but they did not know which one was a placebo. Also, they were allowed to open the drug package. The evaluators scored the sensory similarities in terms of drug appearance, odor, taste, and characteristics. The judgment criteria were as follows: complete consistency corresponded to a score of 10.0 points, comparative consistency was 7.5 points, uncertainty scored 5.0 points, a large difference corresponded to 2.5 points, and complete inconsistency was 0 points. If the single evaluation content was < 5 points, it was considered that there were certain differences between the two samples.

##### Method 2

The evaluators were randomly distributed into two groups, and there were two scenarios in each: 1) both samples were the investigational drug; and 2) one sample was the investigational drug and the other was the placebo. The patients had an equal chance of obtaining either sample in both scenarios. There were a total of 20 evaluators. Each assessed the placebo/investigational drug or the investigational drug/investigational drug simultaneously, and they were allowed to open the packaging. The evaluators considered the similarities between the placebo and investigational drug in terms of packaging, label, strength, drug form, color, odor and taste; they were instructed to determine if the placebo was similar to the investigational drug and whether the placebo could be identified. The judgment criteria were as follows: complete consistency corresponded to a score of 10.0 points, comparative consistency was 7.5 points, uncertainty scored 5.0 points, a large difference corresponded to 2.5 points, and complete inconsistency was 0 points. The 0.05 level was used to determine whether there was a difference in scores between the two groups.

##### Method 3

A three-point test method was used to evaluate the slight differences between the two samples. Evaluation steps: the evaluator was provided with a group of three samples that were coded with a random three-digit number that was different each time. Two of the samples had the same numbers. The evaluators were required to pick out the sample different from the other two, with an equal occurrence rate of the three samples: BAA, ABB, ABA, BAB, AAB, and BBA. The statistical null hypothesis was that it is impossible to distinguish between these two samples based on their characteristic strength. In this case, the probability of correctly identifying an individual sample was *p* = 0.33. The alternative hypothesis was that these two samples could be distinguished based on their characteristic strength. The probability of correctly identifying a control sample, in this case, was *p* > 0.33. Finally, the number of correct responses and the total number of evaluators was statistically analyzed. When the number of correct responses was greater than or equal to the corresponding value at a certain level of the table tested by the three-point test method, the null hypothesis was rejected, and the alternative hypothesis was accepted at the significant level. A total of 36 samples were included, and the cut-off value for a difference between the two samples was 18 at the 0.05 level.

## Results

### Independent Evaluation

 In the independent evaluation of three dosage forms of TCM placebos, there were no significant differences across the groups about the evaluator gender or age, or the time to make a judgment on whether the dispensed drug was the investigational drug or placebo. Three evaluators had taken FFEJJ before the study (all in the placebo group), and four evaluators had taken ZZKZ before evaluation (two in the placebo group). See Tables 5–7 for specific information.

**TABLE 5:** Basic information of patients independently evaluating FFEJJ placebo.

		Placebo (*n* = 10)	Investigational drug (*n* = 10)
Sex	Male	2	2
	Female	8	8
Age (yr)	—	30.67 ± 4.485	38.87 ± 13.967
Evaluation time (seconds)	Mean ± SD	38.87 ± 13.967	23.20 ± 9.414
	Min, max	14, 29	10, 35
Prior FFEJJ use	Yes	3	0
	No	7	10

**TABLE 6:** Basic information of patients independently evaluating ZZKZ placebo.

		Placebo (*n* = 10)	Investigational drug (*n* = 10)
Sex	Male	3	5
	Female	7	5
Age (yr)	—	37.8 ± 17.0	40.7 ± 16.6
Evaluation time (seconds)	Mean ± SD	51.10 ± 27.225	40.90 ± 12.749
	Min, max	30, 61	20, 55
Prior ZZKZ use	Yes	2	2
	No	8	8
Open capsule	Yes	6	8
	No	4	2

**TABLE 7:** Basic information of patients independently evaluating BLWT placebo.

		Placebo (*n* = 10)	Investigational drug (*n* = 10)
Sex	Male	4	5
	Female	6	5
Age (yr)	—	34.4 ± 7.88	39.7 ± 13.57
Evaluation time (seconds)	Mean ± SD	46 ± 56.6	33 ± 34
	Min, max	10, 200	10, 120
Prior BLWT granule use	Yes	0	0
	No	10	10

With regard to the evaluation of the FFEJJ oral placebo simulation effect, when the compound was actually the placebo, three of 10 evaluators judged it as the investigational drug, and when it was actually the investigational drug, seven of 10 evaluators judged both of them as investigational drugs.

Next, we evaluated the placebo simulation effect of ZZKZ capsules. When the compound was actually the placebo, eight of 10 evaluators judged it as the investigational drug; when it was actually the investigational drug, five of 10 evaluators judged it as the investigational drug.

Finally, we evaluated the placebo simulation effect of BLWT granules. When granules were the placebo and investigation drug, respectively, four and seven of 10 evaluators chose it as the investigational drug.

If the cut-off value of the evaluators who judged the investigational drug and placebo as the investigational drug was different at the 0.05 level, it was considered that there was no significant difference between the three different TCM preparations placebos, and the study was appropriately blinded (Tables 4, 8).

**TABLE 8:** Independent evaluation results of evaluators who judged the investigational drug and placebo as the investigational drug.

		No. of evaluators = 20 (50/50 received investigational drug and placebo)	
		Investigational drug	Placebo
FFEJJ	Investigational drug	7	3
	Placebo	3	7
ZZKZ	Investigational drug	5	5
	Placebo	8	2
BLWT	Investigational drug	7	3
	Placebo	4	6

### Comparative Evaluation

#### Method 1

A total of 20 evaluators participated in the placebo evaluation of ZZKZ capsule (10 male and 10 female, age range 27–69 years, mean 44.8 ± 12.2 years). Four evaluators had taken capsules before participating in the evaluation. Eighteen of the 20 evaluators opened the capsules and evaluated the similarity of the compounds. For the ZZKZ capsule similarity evaluation, 13 evaluators correctly identified the placebo (Table 9). If the score in a single evaluation was<5 points, it was identified as a difference. The ZZKZ capsule placebo was generally consistent or indefinitely different in appearance and content characteristics with the investigational drug, with some taste and odor differences.

**TABLE 9:** Comparative evaluation results of ZZKZ for method 1

	Number of evaluators = 20 (50/50 received investigational drug and placebo)			
	Mean	SD	Min	Max
Appearance	8.38	1.81	2.5	10.0
Characteristics	5.25	2.64	2.5	9.0
Odor	4.40	2.83	0.0	10.0
Taste	4.95	2.32	2.5	9.0

#### Method 2

A total of 20 evaluators participated in the comparative evaluation of the placebo simulation effect of ZZKZ capsules; three evaluators in the experimental group had previously taken the capsules. The patients in both groups who participated in the evaluation opened the capsule for discrimination, and the results of appearance, contents, odor, and taste are shown in Table 10. Eight evaluators in the combination group identified the placebo, and nine evaluators in the two investigational drugs group mistook the investigational drug as a placebo. Since psychological effects were ruled out, the investigational drug was not considered to be different from the placebo in appearance, but there were some differences between the two in taste, content traits, and odor.

**TABLE 10:** Comparative evaluation results of ZZKZ for method 2

	No. of evaluators = 10 (investigational drug and placebo)	No. of evaluators = 10 (only assessed the investigational drug)	*p* Values
Appearance (Mean ± SD)	8.650 ± 2.015	8.700 ± 1.207	0.947
Characteristics (Mean ± SD)	3.500 ± 2.134	8.850 ± 1.226	<0.0001
Odor (Mean ± SD)	0.500 ± 1.054	7.550 ± 2.047	<0.0001
Taste (Mean ± SD)	3.600 ± 2.558	6.800 ± 2.394	0.010

#### Method 3

A total of 36 evaluators (16 male, 20 female; age range 18–76 years, mean age 41.72 ± 13.403) participated in the comparative evaluation. None of them had taken the investigational drug before completing the evaluation. The evaluators selected one different sample or two identical samples from each test package regarding appearance, texture, color, odor, and taste. The number of evaluators with correct judgment was less than half of the total, so the comprehensive evaluation result was pass for BLWT granules vs. placebo (Table 11).

**TABLE 11:** Comparative evaluation results of BLWT for method 3

Comparative evaluation content	No. of evaluators who made the correct judgment	Eligibility criteria	Pass or fail
A. Appearance	A = 4	A < 18	Pass
B. Texture	B = 3	B < 18	Pass
C. Color	C = 12	C < 18	Pass
D. Odor	D = 12	D < 18	Pass
E. Taste	E = 16	E < 18	Pass
S. Comprehensive assessment	S = 9.4	S < 18	Pass

## Discussion

An ideal placebo has no active ingredients but is identical to the investigational drug in organoleptic properties. In clinical investigations of western medicine, the active ingredients are clear, so the placebo only needs to use the corresponding excipients (e.g., starch, glucose, etc.,) and the difficulty coefficient is not high (Qi et al., 2008). However, for TCM studies, the Chinese materia medica composition is often several or even dozens of components, and many traditional forms (e.g., decoctions, pills and powders) have special odors and tastes, and ensuring that the placebo is similar can be difficult. There is large variability, which makes placebo preparation even more difficult. Special consideration should be given to the design of a placebo to ensure a good simulation effect.

In actual clinical trials, uniform criteria are often used for the specification, packaging, usage, and dosage of the placebo and investigational drug. The evaluation mainly focuses on whether the placebo has drug activity and whether it is consistent with the investigational drug in terms of appearance, color odor, and taste. Nevertheless, there is no recognized evaluation method for placebo quality evaluation. In addition to avoiding placebo effects, attention should also be paid to nocebo effects during double-blind RCTs. The nocebo effect is defined as a harmful result of patients’ doubts or negative expectations about the treatment (Blasini et al., 2018). Evidence has been found that both the placebo and nocebo effects can substantially affect the efficacy of the drug as well as nondrug treatments (Amanzio et al., 2001; Aslaksen et al., 2015). Therefore, nocebo effects can affect the accurate determination and evaluation of therapeutic drugs. Determining how to scientifically and consistently design placebos and evaluate their simulation effects is particularly important.

Although the objective evaluation method can make the data more objective and reduce the degree of deviation, there are some differences between the objective evaluation method and clinical practice. The patients’ potential psychological factors cannot be 100% simulated, affecting the test results. Also, the standardization and objective quantification of simulation effects still need to be improved (Wang et al., 2003). So, the present study used the artificial evaluation method. By pre-investigating a small number of target evaluators in advance, researchers can predict whether they will distinguish the difference between the placebo and the experimental drug in the actual clinical study. Considering whether the placebo’s simulation effect would be affected if there were simultaneous exposure to two drugs, we provided three different contrast evaluation methods. All of them enabled identifying potential differences between the two samples, and they all have some differences. For the first method, the evaluators knew that there must be a placebo included, so they would pay more attention to finding the differences between the two, and the probability of unblinding was increased. The second method used to deal with this limitation was semi-randomized and evaluated if the two (the investigational drug and placebo, or two investigational drugs) were the same. To make this judgment, both the difference and the consistency, or both of them, had to be considered, making up for the limitation of the first method that only looked for different points. The difference reflects the inconsistencies between the two and the investigational drug, and the consistency expresses the degree of the similarities between the two and the investigational drug. With smaller differences comes higher consistency. The purpose of the evaluation was achieved by using one of the analyses in actual operation. Nevertheless, semi-randomization may also produce some biases, because of the infeasibility of true randomization in a strict sense. The third method adopted complete randomization, eliminateing nonuniformity error, order error caused by the sampling method, and allocation error due to improper allocation. It also reduced subjective and objective biases by combining the blinding approach, thus significantly improving data reliability. We believe that the completely random simulated evaluated better whether the placebo was more consistent with the experimental drug by discussing several comparative evaluation methods. In the actual clinical application, we suggest that the combination of independent and comparative evaluation can be closer to the real double-blind placebo-controlled studies, so as to avoid the placebo and nocebo effects to the greatest extent. Nevertheless, evaluators may have different psychological tendencies and have varying sensitivities to the organoleptic properties of Chinese materia medica so that the manual evaluation method can be affected by subjective factors. Also, in this study, the sample size was small, and more samples are needed for verification.

Concerning the evaluation criteria, there are currently two primary forms. One compares the investigational drug and placebo in terms of shape, color, odor, taste and other aspects to determine whether the differences were significant. The second is to define the value of either artificially, but there is no uniform definition of evaluation criteria cut-off values. This study was mainly based on the relevant literature and statistical indicators in early-stage investigations. There are some doubts about the scientific validity and reliability of this approach, which must be continuously explored in the future.

The literature suggests that evaluators should be asked to indicate whether they think they took the investigational drug or placebo after the trial. The statistical analyses should then estimate and adjust for postrandomization confounding factors that could influence treatment effect according to the causal inference framework to obtain the unbiased efficacy estimate and more easily explain the estimate (Hubbard et al., 2012). At present, there is little evidence that RCTs are actually double-blinded (Hrobjartsson et al., 2007). Our next focus will be to investigate whether it is necessary to specify the evaluation results of the placebo simulation effect along with articles, which should provide better references to readers.

## Conclusion

A blind method is an essential link to a high-quality TCM clinical trial. The simulation effect of the placebo will directly affect whether the blind method can be realized. Evaluation of the TCM placebo’s simulation effect can judge whether the placebo preparation achieves complete consistency in visual attributes, dosage form features and smell/taste attributes. We proposed an independent evaluation method and three comparative evaluation methods, and three dosage forms (oral liquid, capsule and granule) were tested. Regardless of the methods can evaluate well the simulated effect of placebos, in actual clinical trials, we suggest that independent evaluation and comparative evaluation (method 3) should be combined to reflect better whether the placebo is truly blind. This study can provide a new choice for the simulation evaluation of TCM placebo in the future and further improve the quality of TCM clinical trials.

## Data Availability

The original contributions presented in the study are included in the article/Supplementary Material, further inquiries can be directed to the corresponding authors.
